# Supplementary material for: Grb2 binding induces phosphorylation-independent activation of Shp2
Source: Commun Biol. 2021 Apr 1;4:437. doi: 10.1038/s42003-021-01969-7 (PMC8016844; doi:10.1038/s42003-021-01969-7)
Supplement: Supplementary file 2 — Supplementary Information [file 42003_2021_1969_MOESM2_ESM.pdf]

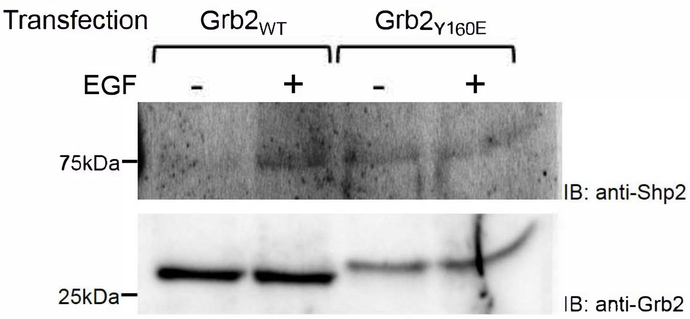

Supplementary Fig. 1: Interaction of Shp2 with monomeric Grb2 in the basal state.
Strep-tagged Grb2 plasmids (dimeric Grb2, Grb2WT; monomeric Grb2, $Grb2_{Y160E}$) were transfected into HEK293T cells. Cells remained serum-starved or were EGF-stimulated. Grb2-Shp2 complexes were pulled down using streptavidin beads and precipitated. Shp2 was immunoblotted using a Shp2 antibody. Both dimeric and monomeric Grb2 can precipitate Shp2 upon EGF stimulation. However, in the absence of EGF stimulation, only monomeric Grb2 ($Grb2_{Y160E}$) can precipitate Shp2. The blot is representative of the results from two independent experiments.

a

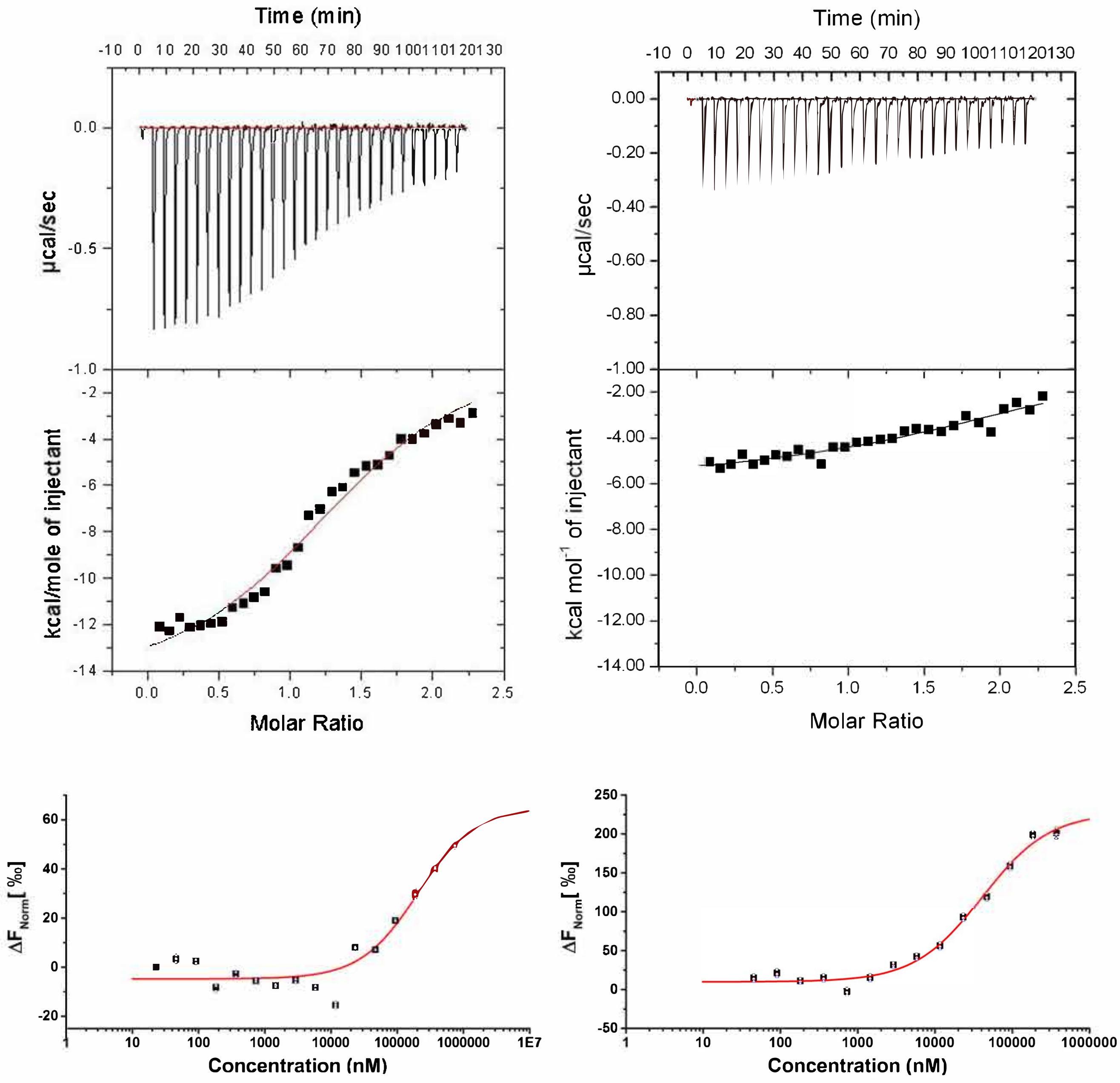

Grb2 pY160: PQQPT{pY}VQALFDF
Grb2 pY209: MFPRN{pY}VTPVNRN

	ITC	MST
Shp2$_{2SH2}$-pY160	20µM	206µM
Shp2$_{2SH2}$-pY209	Weak binding	50µM

b

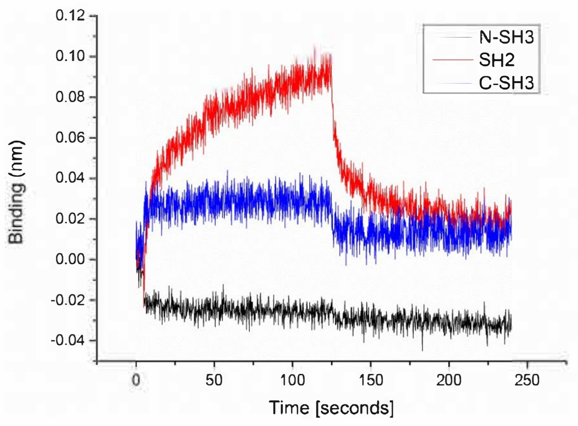

Supplementary Fig. 2: The $Shp2_{2SH2}$ and mGrb2 interaction is independent of phosphorylation of Grb2.

a To confirm that the interaction of Grb2 with Shp2 is independent of phosphorylation, we measured affinities, using both ITC and MST, for the binding of peptides which mimicked the two potential phosphorylated tyrosine residues on Grb2, i.e. including pY160 and pY209, to $Shp2_{2SH2}$. **Left hand side** - for pY160 peptide K_d = 20 μM (using ITC) and 206 μM (MST). **RIght hand side** - for pY209 peptide K_d = too weak to measure (using ITC) and 50 μM (MST) - see **Table below**. In all cases the binding of phosphorylated ligands were approximately two orders of magnitude weaker than the interaction of $Shp2_{2SH2}$ with the non-phosphorylated $Grb2_{Y160E}$ interaction Data from MST are presented as (mean ± SD) of technical triplicates. **b** In order to determine the domain in Grb2 that was responsible for $Shp2_{NSH2}$ binding, GST-tagged $Shp2_{NSH2}$ was immobilised on a BLI sensor and individual Grb2 domains (Black: NSH3, Red: SH2, and Blue: CSH3) were added to identify any interaction. The BLI screen results clearly indicated that the SH2 domain of Grb2 binds to $Shp2_{NSH2}$.

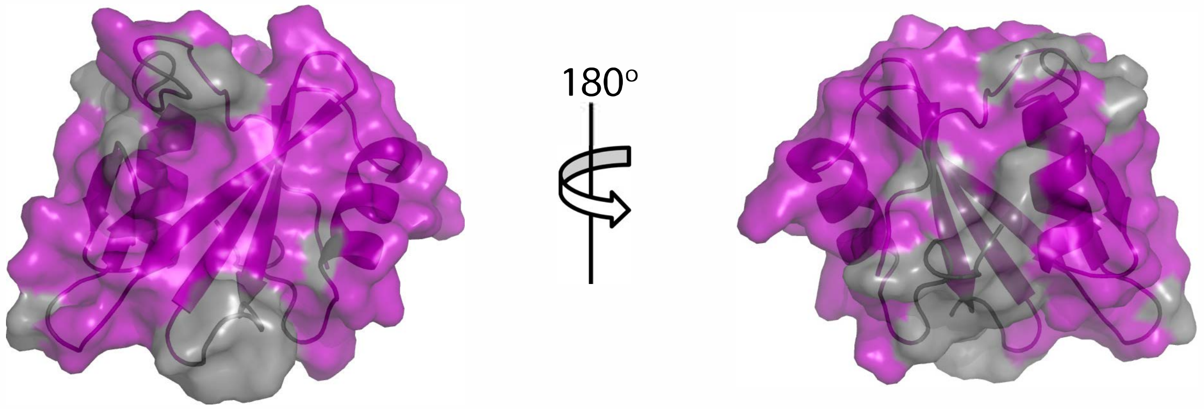

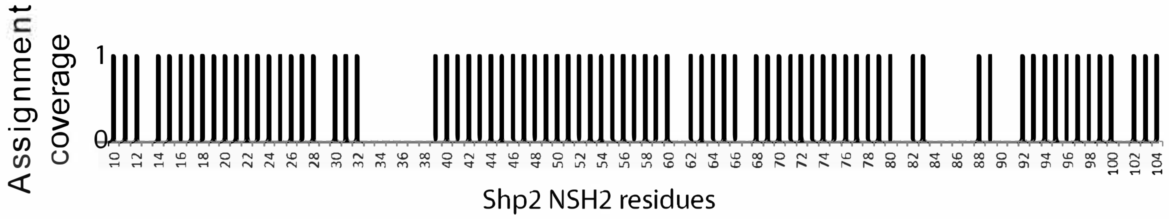

Supplementary Fig. 3: Assignment coverage of Shp2NSH2 domain.
Above: ^{1}H, ^{15}**N** peak assignments mapped onto the space-filling model crystal structure of $Shp2_{NSH2}$ (PDB code 2SHP). The coverage of assigned residues of $Shp2_{NSH2}$ is 81% (assigned redidues in purple)
Below: Plot of assigned residues (black bars) against residue numbers

Supplementary Fugure 4

a

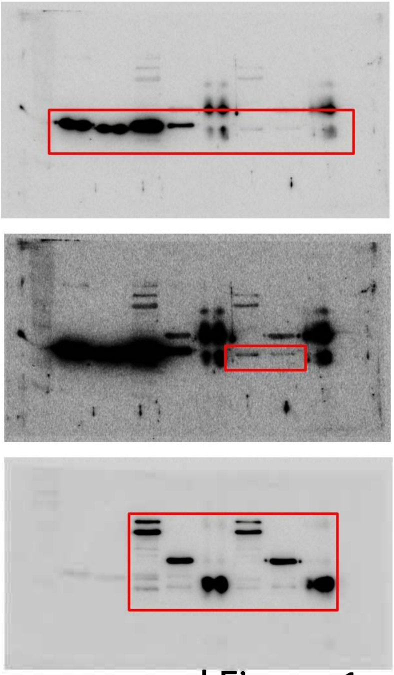

uncropped Figure 1c

b

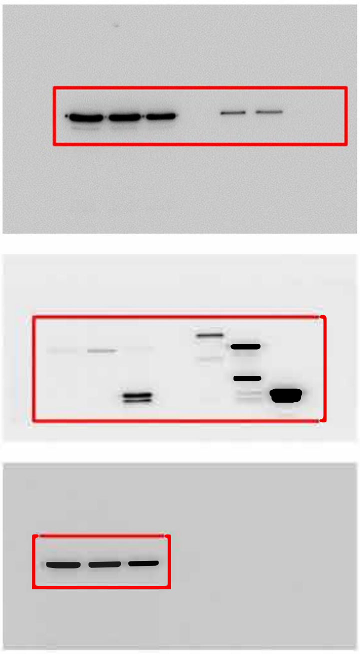

uncropped Figure 1e

c

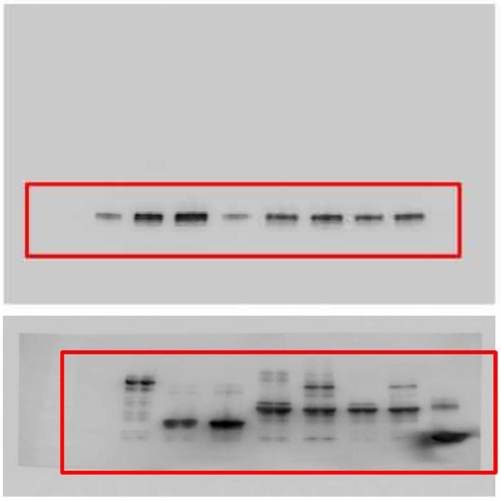

uncropped Figure 2c

d

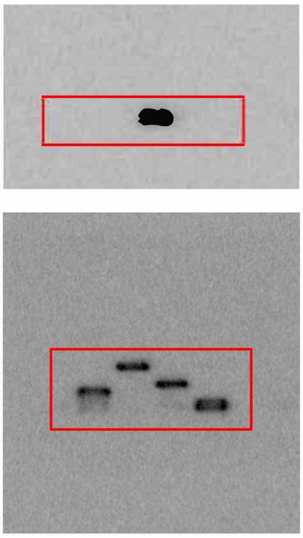

uncropped Figure 3b

e

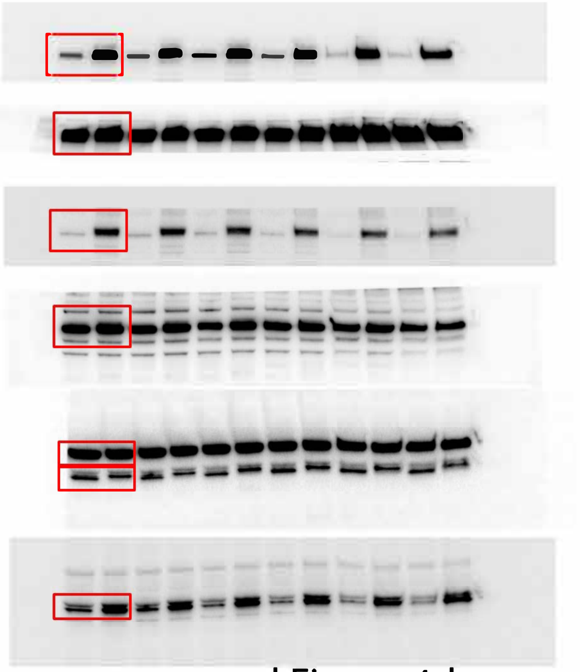

uncropped Figure 4d

Supplementary Fig. 4: Uncropped Western Blots. **a** uncropped blots for Fig. 1c. **b** uncropped blots for Fig. 1e. **c** uncropped blots for Fig. 2c. **d** uncropped blots for Fig. 3b. **e** uncropped blots for Fig. 4d.
