# Supplementary material for: Grb2 binding induces phosphorylation-independent activation of Shp2
Source: Commun Biol. 2021 Apr 1;4:437. doi: 10.1038/s42003-021-01969-7 (PMC8016844; doi:10.1038/s42003-021-01969-7)
Supplement: Supplementary file 3 — Description of Additional Supplementary Files [file 42003_2021_1969_MOESM3_ESM.pdf]

Description of Additional Supplementary Files

File name: Supplementary Data 1

Description: Source data for Fig. 1.

File name: Supplementary Data 2

Description: Source data for Fig. 2.

File name: Supplementary Data 3

Description: Source data for Fig. 3.

File name: Supplementary Data 4

Description: Source data for Fig. 4.

File name: Supplementary Data 5

Description: Source data for Supplementary Fig. 2.

Gaaiho PDF Trial
www.gaaiho.com
