# Supplementary material for: Grb2 binding induces phosphorylation-independent activation of Shp2
Source: Commun Biol. 2021 Apr 1;4:437. doi: 10.1038/s42003-021-01969-7 (PMC8016844; doi:10.1038/s42003-021-01969-7)
Supplement: Supplementary file 9 — Reporting Summary [file 42003_2021_1969_MOESM9_ESM.pdf]

nature research

Corresponding author(s): Chi-Chuan Lin
John E. Ladbury

Last updated by author(s): Feb 15, 2021

Reporting Summary

Nature Research wishes to improve the reproducibility of the work that we publish. This form provides structure for consistency and transparency in reporting. For further information on Nature Research policies, see our Editorial Policies and the Editorial Policy Checklist.

Statistics

For all statistical analyses, confirm that the following items are present in the figure legend, table legend, main text, or Methods section.

n/a	Confirmed	
☐	☒	The exact sample size (*n*) for each experimental group/condition, given as a discrete number and unit of measurement
☐	☒	A statement on whether measurements were taken from distinct samples or whether the same sample was measured repeatedly
☐	☒	The statistical test(s) used AND whether they are one- or two-sided<br>*Only common tests should be described solely by name; describe more complex techniques in the Methods section.*
☒	☐	A description of all covariates tested
☒	☐	A description of any assumptions or corrections, such as tests of normality and adjustment for multiple comparisons
☐	☒	A full description of the statistical parameters including central tendency (e.g. means) or other basic estimates (e.g. regression coefficient) AND variation (e.g. standard deviation) or associated estimates of uncertainty (e.g. confidence intervals)
☒	☐	For null hypothesis testing, the test statistic (e.g. *F*, *t*, *r*) with confidence intervals, effect sizes, degrees of freedom and *P* value noted<br>*Give P values as exact values whenever suitable.*
☒	☐	For Bayesian analysis, information on the choice of priors and Markov chain Monte Carlo settings
☒	☐	For hierarchical and complex designs, identification of the appropriate level for tests and full reporting of outcomes
☒	☐	Estimates of effect sizes (e.g. Cohen's *d*, Pearson's *r*), indicating how they were calculated

Our web collection on statistics for biologists contains articles on many of the points above.

Software and code

Policy information about availability of computer code

Data collection	Nanotemper Analysis software, v.1.5.41, NMRPipe
Data analysis	OriginPro 9.1, CcpNmr Analysis software package, R

For manuscripts utilizing custom algorithms or software that are central to the research but not yet described in published literature, software must be made available to editors and reviewers. We strongly encourage code deposition in a community repository (e.g. GitHub). See the Nature Research guidelines for submitting code & software for further information.

Data

Policy information about availability of data

All manuscripts must include a data availability statement. This statement should provide the following information, where applicable:

- Accession codes, unique identifiers, or web links for publicly available datasets
- A list of figures that have associated raw data
- A description of any restrictions on data availability

The authors declare that all data supporting the findings of this study are available within the article and its supplementary information files.

Field-specific reporting

Life sciences study design

All studies must disclose on these points even when the disclosure is negative.

Sample size	MST data was carried out as 2-3 technical replicates; FLIM imaging was carried out as 2 biological replicates; pulldown and immunoprecipitation experoments were carried out as 3 biological replicates. Phosphatase assays were carried out as 2-3 biological replicates. MTT proliferation assay was carried out as 2 biological replicates.
Data exclusions	n/a
Replication	All attempts to reproduce data were successful.
Randomization	Randomization was not required.
Blinding	Blinding was not required in this study.

Reporting for specific materials, systems and methods

We require information from authors about some types of materials, experimental systems and methods used in many studies. Here, indicate whether each material, system or method listed is relevant to your study. If you are not sure if a list item applies to your research, read the appropriate section before selecting a response.

Materials & experimental systems

n/a	Involved in the study	
☐	☒	Antibodies
☐	☒	Eukaryotic cell lines
☒	☐	Palaeontology and archaeology
☒	☐	Animals and other organisms
☒	☐	Human research participants
☒	☐	Clinical data
☒	☐	Dual use research of concern

Methods

n/a	Involved in the study	
☒	☐	ChIP-seq
☒	☐	Flow cytometry
☒	☐	MRI-based neuroimaging

Antibodies

Antibodies used	Anti-Grb2 (sc-8034) were purchased from Santa Cruz Biotechnology. Anti-GST (2624), anti-GFP (2956), anti-Tubulin (2146), anti-pEGFR pY1173 (2244), anti-EGFR (4267), anti-Shp2 pY580 (3754), anti-pErk1/2 (4370), and anti-Erk1/2 (9101) were from Cell Signaling Technology. Anti-RFP (A00682) was purchased from Genscript. Anti-6xHis (631210) was purchased from Takara. Anti-Shp2 antibodies were purchased from Santa Cruz Biotechnology, Cell Signal Technology, Sigma or Abcam. Anti-Grb2 pY160 was synthesized from Genscript.
Validation	All antibodies apart form Anti-Grb2 pY160 were validated by the manufactures. Anti-Grb2 pY160 was generated by Genscript and used in our previous study (Z. Ahmed, Z. Timsah, K. M. Suen, N. P. Cook, G. R. Lee IV, C. –C. Lin, M. Gagea, A. A. A. Marti, J. E. Ladbury, Grb2 monomer–dimer equilibrium determines normal versus oncogenic function. Nat. Commun. 6, 8007 (2015)).

Eukaryotic cell lines

Policy information about cell lines

Cell line source(s)	Cell lines were purchased from ATCC
Authentication	Cell lines from ATCC have been thoroughly tested and authenticated
Mycoplasma contamination	Cell lines tested negative for mycoplasma contamination
Commonly misidentified lines (See ICLAC register)	n/a
